# Impact of a Purified Microbiome Therapeutic on Abundance of Antimicrobial Resistance Genes in Patients With Recurrent *Clostridioides difficile* Infection

**DOI:** 10.1093/cid/ciad636

**Published:** 2023-10-12

**Authors:** Timothy J Straub, Mary-Jane Lombardo, Jessica A Bryant, Liyang Diao, Thomas P Lodise, Daniel E Freedberg, Jennifer R Wortman, Kevin D Litcofsky, Brooke R Hasson, Barbara H McGovern, Christopher B Ford, Matthew R Henn

**Affiliations:** Seres Therapeutics, Cambridge, Massachusetts, USA; Seres Therapeutics, Cambridge, Massachusetts, USA; Seres Therapeutics, Cambridge, Massachusetts, USA; Seres Therapeutics, Cambridge, Massachusetts, USA; Albany College of Pharmacy and Health Sciences, Albany, New York, USA; Division of Digestive and Liver Diseases, Columbia University Irving Medical Center–New York Presbyterian Hospital, New York, New York, USA; Seres Therapeutics, Cambridge, Massachusetts, USA; Seres Therapeutics, Cambridge, Massachusetts, USA; Seres Therapeutics, Cambridge, Massachusetts, USA; Seres Therapeutics, Cambridge, Massachusetts, USA; Seres Therapeutics, Cambridge, Massachusetts, USA; Seres Therapeutics, Cambridge, Massachusetts, USA

**Keywords:** microbiome therapeutics, VOWST, SER-109, *Clostridioides difficile* infection, antimicrobial resistance

## Abstract

**Background:**

The gastrointestinal microbiota is an important line of defense against colonization with antimicrobial resistant (AR) bacteria. In this post hoc analysis of the phase 3 ECOSPOR III trial, we assessed impact of a microbiota-based oral therapeutic (fecal microbiota spores, live; VOWST Oral Spores [VOS], formerly SER-109]; Seres Therapeutics) compared with placebo, on AR gene (ARG) abundance in patients with recurrent *Clostridioides difficile* infection (rCDI).

**Methods:**

Adults with rCDI were randomized to receive VOS or placebo orally for 3 days following standard-of-care antibiotics. ARG and taxonomic profiles were generated using whole metagenomic sequencing of stool at baseline and weeks 1, 2, 8, and 24 posttreatment.

**Results:**

Baseline (n = 151) and serial posttreatment stool samples collected through 24 weeks (total N = 472) from 182 patients (59.9% female; mean age: 65.5 years) in ECOSPOR III as well as 68 stool samples obtained at a single time point from a healthy cohort were analyzed. Baseline ARG abundance was similar between arms and significantly elevated versus the healthy cohort. By week 1, there was a greater decline in ARG abundance in VOS versus placebo (*P* = .003) in association with marked decline of Proteobacteria and repletion of spore-forming Firmicutes, as compared with baseline. We observed abundance of Proteobacteria and non–spore-forming Firmicutes were associated with ARG abundance, while spore-forming Firmicutes abundance was negatively associated.

**Conclusions:**

This proof-of-concept analysis suggests that microbiome remodeling with Firmicutes spores may be a potential novel approach to reduce ARG colonization in the gastrointestinal tract.

## Teaser

In a post hoc analysis of ECOSPOR III study patients with recurrent *Clostridioides difficile* infection, VOWST Oral Spores rapidly and significantly reduced antimicrobial resistance genes compared with placebo, supporting microbiome therapeutics as a potential novel approach to combatting antimicrobial resistance.

Antimicrobial resistance (AMR) is responsible for an estimated 4.9 million deaths annually worldwide [1]. Yet, this urgent healthcare crisis has worsened with the increased prevalence of emergence of extended-spectrum beta-lactamase–producing Enterobacterales and carbapenem-resistant *Enterobacteriaceae* [2, 3]. Due to the inherent risk of emerging resistance, there is a general reluctance to use new antibacterials, creating a roadblock for research and drug development [4, 5]. New therapeutic approaches with different mechanisms of action are needed to address this global problem.

The gastrointestinal (GI) tract is a potential reservoir harboring multidrug-resistant bacteria, which brings to bear new ideas on how to confront this healthcare crisis [6]. The GI microbiome plays a key role in colonization resistance against AMR pathogens and *Clostridioides difficile* [7]. Numerous epidemiologic studies have demonstrated that GI colonization and pathogen domination frequently precede infection with AMR pathogens [8], such as vancomycin-resistant enterococcal bacteria in adults undergoing allogeneic hematopoietic stem cell transplantation [9]. The major risk factor for colonization is receipt of antimicrobials, which disrupt the GI microbiome and lead to low microbial diversity and loss of colonization resistance [10, 11]. Depletion of gram-positive bacteria within the Firmicutes phylum (newly named Bacillota [12]) leads to a loss of microbe-associated metabolites important for protection against bacterial pathogens [13]. Specifically, spore-forming Firmicutes, such as members of *Clostridiales* (eg, *Lachnospiraceae*, *Ruminococcaceae*), are thought to play an important role in bile acid metabolism pathways [14]. Loss of abundance of Firmicutes bacteria is also associated with reciprocal expansion of gram-negative bacteria within the Proteobacteria phylum (newly named Pseudomonadota [12]), which normally make up less than 1% to approximately 2% of the healthy GI microbiome [15, 16]. Since gram-negative members of the Proteobacteria phylum (eg, *Klebsiella*, *Pseudomonas*) are known to harbor AMR genes (ARGs) [17], patients with recurrent *C. difficile* (rCDI) are at high risk of colonization with drug-resistant bacteria due to repeated antibiotic exposure.

In a phase 3 randomized trial of adults with a history of rCDI (ECOSPOR III), fecal microbiota spores, live (VOWST; formerly SER-109 and hereafter referred to as VOS for VOWST Oral Spores; Seres Therapeutics), an orally administered microbiome therapeutic composed of Firmicutes spores, significantly reduced CDI recurrence compared with placebo (12% in the VOS group and 40% in the placebo group) following standard-of-care antibiotics [18]. In an exploratory analysis of the phase 3 data, VOS treatment also led to significant increases in the relative abundance of Firmicutes bacteria and marked reciprocal reductions in Proteobacteria as compared with placebo (Bryant, 2023, unpublished data). In light of the microbiome remodeling observed, we postulated that VOS may reduce the reservoir of drug-resistant microbes and their associated ARGs. In this post hoc analysis, we assessed the impact of VOS, compared with placebo, on ARG abundance, as well as how the taxonomic composition of the microbiome correlated with the abundance of ARGs. As a reference, we also show baseline comparisons to a healthy cohort.

## METHODS

### Study Design and Patients

The study design and methods are described in detail elsewhere [18, 19]. ECOSPOR III was a multicenter, double-blind, phase 3 study conducted at 56 US and Canadian sites from July 2017 to September 2020. The protocol and amendments were approved by institutional review boards and all participants provided written informed consent at screening. The study included adults 18 years of age or older with 3 or more CDI episodes within 12 months, inclusive of the qualifying acute episode, which was defined as follows: (1) 3 or more unformed bowel movements over 2 consecutive days, (2) a positive *C. difficile* toxin test by enzyme immunoassay or reflex cytotoxicity neutralization assay, and (3) symptom resolution after 10–21 days of standard-of-care antibiotics.

Patients were stratified by age (<65 or ≥65 years) and antibiotic for their qualifying episode (ie, vancomycin or fidaxomicin) and randomly assigned 1:1 to VOS (∼3 × 10^7^ spore colony-forming units/d) or matching placebo administered as 4 oral capsules once daily over 3 consecutive days [18]. Patients consumed 10 ounces of magnesium citrate or 250 mL of polyethylene glycol 1 day prior to treatment initiation, after completing antibiotics.

### Sample Collection, Sequencing, and Processing

Additional details on sample collection, sequencing, and processing can be found in the Supplementary Methods. Briefly, baseline stool samples were collected within 3 days following cessation of antibiotics, prior to bowel cleanse, and posttreatment stool samples were collected at weeks 1, 2, 8, and 24.

DNA was extracted from stool samples and libraries were prepared and sequenced to a target depth of 10 Gb per sample. Sample whole metagenomic sequencing data were processed per standard guidelines.

Taxonomy profiling was performed using MetaPhlAn2 [20] with a proprietary genomic database [18], to produce species-level relative abundance profiles. ARG profiling was performed using ShortBRED [21] and a protein marker database derived from the Comprehensive Antibiotic Resistance Database (CARD) [22]. ARGs were detected with a 90% identity cutoff to the marker database. Output was per-sample normalized counts per ARG, represented in reads per kilobase million (RPKM). The ARG counts were summarized into drug-class resistances and resistance mechanisms based on CARD ontology. Total ARG abundance (in RPKM) was calculated as the sum of normalized counts across all ARGs for a given sample. See Supplementary Material for further details. Information regarding the healthy cohort is provided in the Supplementary Material.

### Endpoints

Prespecified clinical and microbiome endpoints are reported in Feuerstadt et al [18]. In this post hoc analysis, we included all patients who were randomized, received any amount of study drug, and had at least 1 evaluable baseline and at least 1 evaluable posttreatment stool sample. Due to major protocol deviations (eg, use of prohibited medications, inclusion/exclusion criteria not met), 56 samples from 16 patients (8 from VOS, 8 from placebo) were excluded from analysis.

### Statistical Analysis

All statistical analyses were calculated in R version 3.6.0 (R Foundation for Statistical Computing). Linear mixed models were run using lme4 [23] and lmerTest [24]. To assess treatment differences, difference-in-difference linear mixed models were used to account for patient variability and baseline resistance profiles. Time and treatment arm (and their interaction) were included as fixed effects. Antibiotic (ie, vancomycin or fidaxomicin) was an additional fixed effect (ie, covariate), and patient was a random effect. A different model was performed for each of the following response variables: log-transformed normalized total ARG abundance, log-transformed abundance of each antibiotic drug class, log-transformed abundance of each resistant mechanism, and the relative abundance of specific taxa in the taxonomic composition analyses.

Taxon–ARG correlations were obtained using linear mixed models with patient as a random effect, taxa relative abundance as a fixed effect, and normalized ARG abundance as the response variable (see Supplementary Figure 4). Each taxon–ARG category was modeled independently and reported *P* values were corrected for false discovery rate using Benjamini-Hochberg [25].

## RESULTS

### Demographics

A total of 182 patients were randomized (59.9% female; mean age: 65.5 years). All patients had a minimum of 3 episodes of CDI and the majority (73.1%) were treated with vancomycin for the qualifying acute CDI recurrence. A full description of patient demographics and baseline characteristics is found elsewhere [18, 19]. Baseline stool samples (n = 151) and serial posttreatment samples collected through 24 weeks (total N = 472) were available for analysis. There were a greater number of samples available for analysis in the VOS versus the placebo arm due to the higher discontinuation rate in placebo patients, mainly due to greater on-study CDI recurrences.

### Taxonomic Characteristics at Baseline

**Figure 1.**
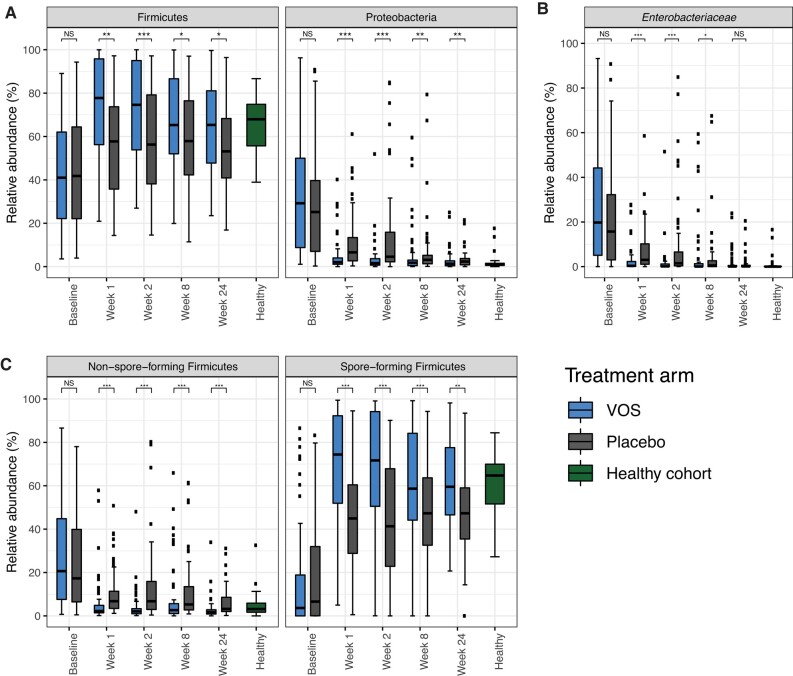
Relative abundance of Proteobacteria and Firmicutes in ECOSPOR III and in a healthy cohort. *A*, Relative abundance of the Firmicutes and Proteobacteria phyla over time in both study arms. *B*, Relative abundance of *Enterobacteriaceae* over time in both study arms*. C*, Relative abundance of non–spore-forming and spore-forming Firmicutes over time in both study arms. The healthy cohort not enrolled in ECOSPOR III is displayed for reference. NS: *P* ≥ .05; *P < .05; **P < .01; ***P < .001. Abbreviations: NS, not significant; VOS, VOWST Oral Spores.

At baseline, the abundance of Proteobacteria was significantly elevated, at a median of 29% and 25% abundance for VOS and placebo arms, respectively (Supplementary Table 1, Figure 1*A*), approximately 27 and 23 times higher median abundance in the VOS and placebo arms, respectively, compared with the healthy cohort, which had minimal representation of Proteobacteria (ie, median: 1.1%; interquartile range: 1.0%). In contrast, the abundance of the Firmicutes phylum at baseline was depleted in both treatment arms when compared with the healthy cohort (Supplementary Table 1, Figure 1*A*). Within the Firmicutes phylum, non–spore-forming Firmicutes, which include many clinically relevant pathogens (eg, *Enterococcus, Streptococcus*, *Staphylococcus*), were greater in abundance than spore-forming Firmicutes (eg, *Lachnospiraceae*, *Ruminococcaceae*, *Clostridiaceae*) in both treatment arms compared with the healthy cohort (Supplementary Table 2, Figure 1*B*).

### Posttreatment Taxonomic Changes

Patients treated with VOS versus placebo had increased levels of Firmicutes and decreased levels of Proteobacteria at all posttreatment time points (Supplementary Table 1, Figure 1*A*). VOS treatment was associated with rapid and significant declines in the Proteobacteria family *Enterobacteriaceae* compared with placebo at weeks 1 and 2, with an overall decline from a median of 20% at baseline to 0.4% at week 1 and 0.2% at Week 2 (Figure 1*B*). In the placebo arm, *Enterobacteriaceae* abundance decreased slowly after antibiotic discontinuation. Compared with placebo, VOS treatment led to significant increases in the spore-forming Firmicutes, with reciprocal declines in non–spore-forming Firmicutes, across all posttreatment time points (Figure 1*C*).

### Antibiotic Resistance Gene Abundance

**Figure 2.**
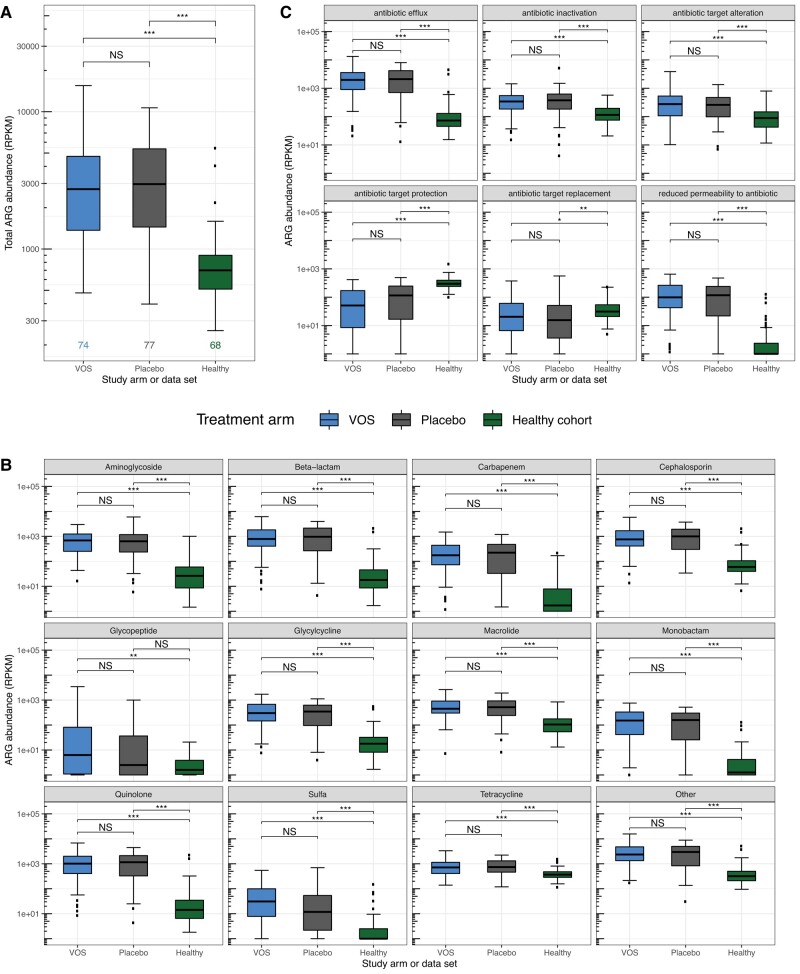
The abundance of ARGs at baseline in ECOSPOR III compared with a healthy cohort. *A*, Total ARG abundance (normalized for gene length and sample sequencing depth to RPKM) at baseline in patients in both treatment arms of ECOSPOR III as compared with the healthy cohort. *B*, The abundance of ARGs conferring resistance to various antibiotic classes at baseline in patients in both treatment arms as compared with the healthy cohort. *C*, The abundance of ARGs with various resistance mechanisms in patients in both treatment arms as compared with the healthy cohort. *P* values are reported for all pairwise comparisons of VOS and placebo treatment arms and the healthy cohort using Wilcoxon rank-sum tests with multiple hypothesis correction. NS: *P* ≥ .05; *P < .05; **P < .01; ***P < .001. Abbreviations: ARG, antimicrobial resistance gene; NS, not significant; RPKM, reads per kilobase million; VOS, VOWST Oral Spores.

At baseline, total ARG abundance (Figure 2*A*) and specific ARGs abundance by drug classes identified by CARD (Figure 2*B*) were significantly elevated for both treatment arms compared with the healthy cohort for most antibiotic classes, but comparable between VOS and placebo. Supplementary Figure 1 displays baseline abundance of ARGs conferring resistance across all antibiotic drug classes in CARD.

The abundance of ARGs represented by different resistance mechanisms was balanced across both treatment arms at baseline (Figure 2*C*). However, in general, patients in both arms had a distinct distribution of resistance mechanisms compared with the healthy cohort. For example, efflux pumps, which confer broad resistance to quinolones, beta-lactams, cephalosporins, macrolides, and other antibiotics, were highly represented in VOS and placebo arms compared with the healthy cohort (Figure 2*C*). In contrast, the healthy cohort had elevated abundances of ARGs with resistance mechanisms of antibiotic target protection and antibiotic target replacement, which mainly confer resistance to tetracyclines and sulfonamides, respectively.

**Figure 3.**
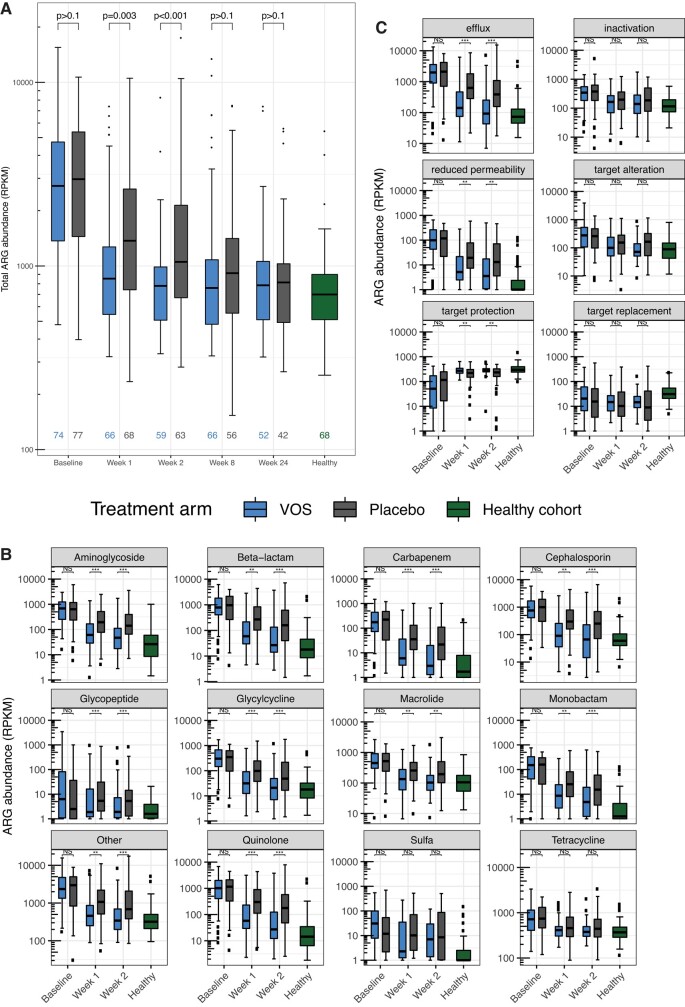
Dynamics of ARGs over time in VOS and placebo study arms. *A*, Total ARG abundance (in RPKM) through week 24 for VOS (blue) and placebo (gray). The healthy cohort (in green) not enrolled in ECOSPOR III is shown as reference only. *B*, The abundance of ARGs conferring resistance to various antibiotic classes through week 2 for study arms. *C*, The abundance of ARGs with various mechanisms of resistance through week 2 for study arms. *P* values are reported for VOS versus placebo at each time point using a difference-in-difference linear mixed model. NS: *P* ≥ .05; *P < .05; **P < .01; ***P < .001. Abbreviations: ARG, antimicrobial resistance gene; NS, not significant; RPKM, reads per kilobase million; VOS, VOWST Oral Spores.

In both treatment arms, total ARG abundance significantly decreased from baseline by week 1 and through week 24 (Figure 3*A*, Supplementary Table 3). However, the magnitude of decline was significantly greater in the VOS arm compared with placebo at weeks 1 and 2.

Compared with placebo, VOS-treated patients had significantly lower abundances of ARGs by specific antibiotic drug class at weeks 1 and 2 (Figure 3*B*). Notably, resistance to glycopeptides (eg, vancomycin) at baseline was elevated in patients in both treatment arms compared with the healthy cohort. At all posttreatment time points, including at week 24, resistance to glycopeptides was significantly lower in the VOS arm compared with placebo (Supplementary Figure 2, Supplementary Table 4).

Compared with placebo, VOS-treated patients had significantly lower “antibiotic efflux” and “reduced permeability to antibiotic” at weeks 1 and 2 (Figure 3*C*). VOS-treated patients also had significantly elevated levels of “antibiotic target protection” compared with placebo at weeks 1 and 2.

Comparisons of posttreatment taxonomic changes in both the VOS and placebo arms in ECOSPOR III relative to the healthy cohort reference are provided in the Supplementary Tables 1–4.

### Correlation of Taxonomic Classification With Antibiotic Resistance Gene Abundance

**Figure 4.**
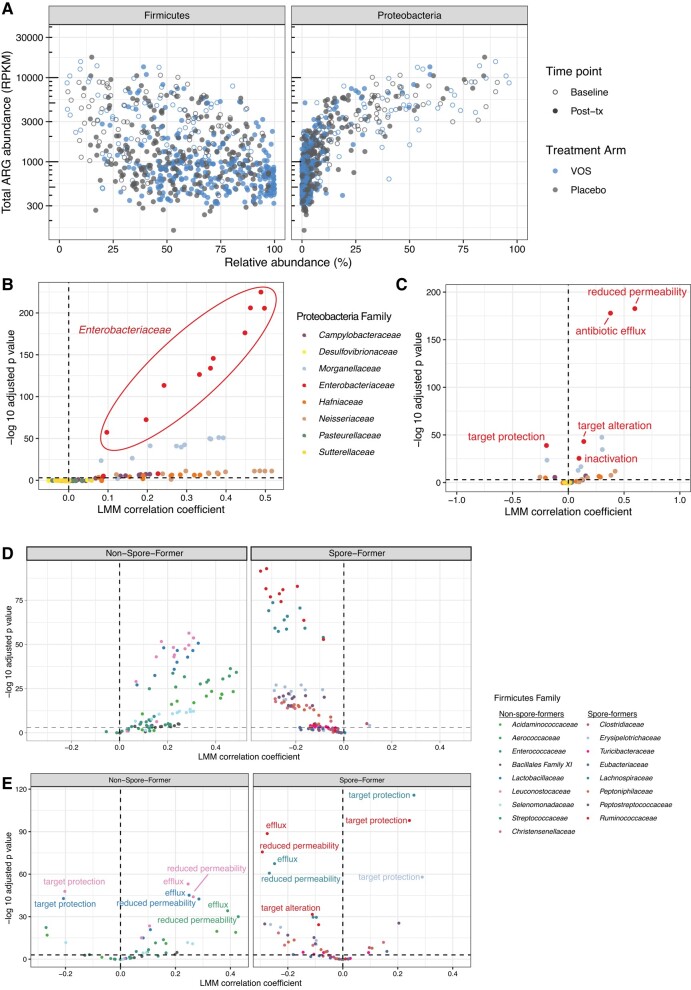
Correlations of ARGs with taxonomic abundance in ECOSPOR III. *A*, Correlation of total ARG abundance with abundance of Firmicutes and Proteobacteria. *B*, Linear mixed-model results correlating abundance of ARGs of different antibiotic classes with relative abundance of Proteobacteria families. *C*, Linear mixed-model results correlating abundance of ARGs of different resistance mechanisms with relative abundance of Proteobacteria families. *D*, Linear mixed-model results correlating abundance of ARGs of different antibiotic classes with relative abundance of non–spore-forming and spore-forming Firmicutes families. *E*, Linear mixed-model results correlating abundance of ARGs of different resistance mechanisms with relative abundance of non–spore-forming and spore-forming Firmicutes families. Abbreviations: ARG, antimicrobial resistance gene; LMM, linear mixed model; Post-tx, posttreatment; VOS, VOWST Oral Spores.

Overall Proteobacteria relative abundance was positively correlated with total ARG abundance across all time points (*P* < .001; Figure 4*A*). Combining samples across all time points (from baseline through week 24), the *Enterobacteriaceae* family was strongly correlated with ARGs conferring resistance to numerous antibiotic drug classes, including quinolones, beta-lactams, and carbapenems (Figure 4*B*, circled points).

In contrast, overall Firmicutes relative abundance was negatively correlated with total ARG abundance (Figure 4*A*, left), but there were distinct and reciprocal associations of the types of Firmicutes with ARG abundance. Specifically, there was a positive association of ARGs with non–spore-forming Firmicutes. In contrast, spore-forming Firmicutes, which comprise VOS, were negatively associated with abundance of ARGs of various drug classes (Figure 4*D*). These differing associations of spore- and non–spore-forming Firmicutes with ARGs were consistent when independently analyzing each treatment arm (Supplementary Figure 3).

Notably, the relative abundance of *Enterobacteriaceae* was highly correlated with antibiotic efflux and reduced permeability to antibiotic (Figure 4*C*), which mainly conferred resistance to tetracycline, quinolones, cephalosporins, carbapenems, and beta-lactams, among other drug classes. While relative abundance of non–spore-forming Firmicutes families had positive correlations with abundances of antibiotic efflux and reduced permeability to antibiotic (Figure 4*E*), relative abundance of spore-forming Firmicutes families was negatively correlated with both of those mechanisms (Figure 4*E*). Spore-forming Firmicutes relative abundances were also positively correlated with antibiotic target protection.

## DISCUSSION

Patients with rCDI are at high risk of harboring ARGs [26], as demonstrated by the wide spectrum of resistance observed at baseline across both intervention and placebo arms in this phase 3 trial. In this proof-of-concept post hoc analysis, when compared with placebo, VOS treatment was associated with an accelerated reduction of ARG abundance, as illustrated by a significantly greater reduction in ARGs at early posttreatment time points. Furthermore, this effect was likely achieved through microbiome remodeling with broad compositional changes across 2 phyla that were either dominant (ie, Proteobacteria) or depleted (ie, Firmicutes) following antibiotic exposure. The placebo comparison and the balanced distribution of ARGs across both arms at baseline made this an ideal study population to assess the impact of VOS on ARGs in this hypothesis-generating analysis. Finally, the observation that spore-forming Firmicutes are negatively associated with the abundance of ARGs has been underappreciated and may provide a new avenue of providing commensals important to human health, while also reducing the abundance of ARGs in the GI microbiome.

At baseline, patients in both arms had evidence of a distinct distribution of resistance mechanisms compared with the healthy cohort. Glycopeptide resistance at baseline was notably elevated in both treatment arms compared with the healthy cohort, which is consistent with the highly prevalent use of vancomycin in these study patients treated for rCDI. These data may be informative to physicians who have often used this antibiotic cyclically for multiple episodes of CDI. In addition, efflux pump genes, which confer broad resistance to multiple widely used, broad-spectrum drug classes for serious infections (eg, fluoroquinolones) [27], were highly represented in both arms at baseline compared with the healthy cohort. In contrast, the healthy cohort had elevated abundances of ARGs that are commonly seen in large epidemiologic population studies, conferring resistance to more narrow-spectrum antibiotics, including tetracyclines and sulfonamides, respectively. This observation is consistent with the ubiquitous presence of tetracycline-resistance proteins in the environment and the human GI microbiome, as shown in several international studies [28, 29]. However, after VOS administration, ARG abundance was significantly reduced compared with placebo as early as week 1. In vulnerable patient populations known to be at increased risk of microbial translocation across the GI tract, such temporal differences may be potentially meaningful, as mortality rates are higher with drug-resistant infections.

Recognition of the GI tract as a reservoir for multidrug-resistant bacteria, amenable to remodeling through microbiome therapeutics, creates a potential new model for combating drug resistance. In the disrupted microbiome of patients with rCDI, Proteobacteria, such as *Klebsiella*, *Escherichia*, and *Pseudomonas*, are unusually abundant compared with the healthy microbiome [30], as observed in our healthy cohort. Notably, the relative abundance of the family *Enterobacteriaceae*, which was highly correlated with resistance to several important antibiotic classes and key resistance mechanisms, was reduced after VOS dosing compared with placebo. In the VOS arm, we also saw a decline in non–spore-forming Firmicutes, such as the enterococci, common gut-seeded, hospital-acquired pathogens [31]. Coincident with these downward shifts in abundance was a reciprocal increase in gram-positive spore-forming bacteria that comprise VOS, which were negatively correlated with drug resistance. Thus, the observed reduction in ARGs is likely due to restructuring of the microbiome towards a healthy state where Proteobacteria become a minority population driven by engraftment of spore-forming Firmicutes that are less apt to harbor ARGs. This inverse association of spore-forming Firmicutes with abundance of ARGs supports the hypothesis that treatment with VOS does not contribute to the emergence of drug resistance, in contrast to antibiotics. In addition, VOS may offer additional benefits beyond full-spectrum microbiota products, which have the potential to contain ARGs simply by the inherent nature of the product.

There are several limitations to these analyses. As in any post hoc analysis, there may be underlying differences in the patient populations or biases that may account for our observations that are not inherently apparent. The hypothesis that microbiome restoration may reduce ARG abundance through microbiome remodeling would need to be tested in a prospective clinical trial. In addition, it is unclear whether the specific strains of spore-forming Firmicutes that comprise VOS or the microbiome remodeling and subsequent expansion of all spore-forming Firmicutes following VOS treatment led to the observed reduction in ARGs. The tools used to detect strain-level differences in metagenomic data are still being developed [32]. Therefore, we opted to apply a commonly used, validated species-detection methodology, which limits our ability to directly answer this question. Since this was a post hoc analysis, we included a healthy cohort as a reference to provide additional context to the abundance of ARGs across drug classes and resistance mechanisms at baseline. In addition, these characteristics were comparable at baseline between VOS and placebo recipients, which strengthens the implications of these data. Also, since stool samples were not collected between weeks 2 and 8, we are limited in our ability to discern dynamic changes between VOS and placebo recipients for approximately a 6-week period of time. Our observations that VOS reduces ARGs are consistent with other published data using fecal microbiota transplantation (FMT) and FMT-like drugs [33]. However, whole-stool products that include gram-negative bacteria have the potential to carry Proteobacteria ARGs, despite donor screening for carriage of drug-resistant bacteria [34].

In conclusion, in this post hoc analysis of a randomized trial of patients at high risk of harboring drug-resistant bacteria, VOS more rapidly reduced the abundance of ARGs, as compared with placebo, and attained an ARG profile similar to a healthy cohort. These changes in ARGs were durable through 24 weeks posttreatment and were observed in association with remodeling of the GI microbiome. This proof-of-concept analysis suggests the feasibility of using microbiome therapeutics as a novel approach to address the urgent problem of reducing drug resistance, by leveraging the beneficial characteristics of spore-forming Firmicutes that may be less likely to harbor ARGs. Future trials should evaluate whether cultivated microbiome therapeutics may prevent carriage of drug-resistant bacteria in the gut and subsequent infections in vulnerable populations.

## Supplementary Data

Supplementary materials are available at *Clinical Infectious Diseases* online. Consisting of data provided by the authors to benefit the reader, the posted materials are not copyedited and are the sole responsibility of the authors, so questions or comments should be addressed to the corresponding author.

## Supplementary Material

ciad636_Supplementary_Data

## Notes

***Author Contributions.*** M. R. H., J. W., and C. B. F. conceptualized the study design. T. J. S., L. D., and J. A. B. collected the data. M. R. H., J. W., K. D. L., C. B. F., and M.-J. L. reviewed the data. T. J. S. and L. D. performed the statistical analysis and the investigation. T. J. S., B. H. M., and B. R. H. wrote the first draft of the manuscript. T. J. S. and L. D. directly accessed the data and verified the underlying data reported in the manuscript. All authors contributed to the interpretation of the data and reviewing and revising of the manuscript and agree with its content and conclusions.

***Acknowledgments.*** The authors thank Meghan Chafee for her thorough review of the manuscript and editorial assistance and Sean Sykes for helpful assistance in data analyses. They thank the ECOSPOR III study investigators who made this trial possible. They are indebted to all the patients who participated in the ECOSPOR III study.

***Financial support*.** This work was supported by Seres Therapeutics.
