# Supplementary material for: Impact of a Purified Microbiome Therapeutic on Abundance of Antimicrobial Resistance Genes in Patients With Recurrent Clostridioides difficile Infection
Source: Clin Infect Dis. 2023 Oct 12;78(4):833–41. doi: 10.1093/cid/ciad636 (PMC11006105; doi:10.1093/cid/ciad636)
Supplement: ciad636_Supplementary_Data [file ciad636_supplementary_data.docx]

***Supplementary Online Content***

**SUPPLEMENTARY METHODS**

**Sample Collection, Sequencing, and Processing**

Samples were collected using a commode collection system (Fisherbrand Commode Specimen Collection System, 02-544-208, Fisher Scientific, Hampton, NH) and shipped on frozen gel packs to a central laboratory where they were homogenized, suspended in 95% ethanol, aliquoted, and then immediately frozen at -80°C.

DNA was extracted from stool samples using a modified version of the MagBind Universal Pathogen 96 kit (Omega bio-tek, Norcross, GA). Libraries were prepared using the Nextera DNA Flex kit (Illumina, San Diego, CA) and sequenced with an Illumina NovaSeq 6000 platform to a target depth of 10 gigabases per sample. Sample WMS data were processed per standard HMP-2 data processing guidelines (<https://hmpdacc.org/hmp/resources/>).

## Sequence Data Processing

Sample WMS data were processed per standard HMP-2 data processing guidelines (<https://hmpdacc.org/hmp/resources/>). Read pair deduplication was implemented using a custom Python function to remove polymerase chain reaction and sequencing artifacts. Adapter sequences and low-quality sequence data was trimmed using trimmomatic.[1] Finally, reads were aligned to human and artificial reference sequences using bowtie2 [2] to remove unwanted sequence data (*e.g.*, human DNA, artificial DNA). Note, read subsampling was performed for taxonomic profiling, but not for ARG profiling.

## Microbiome Taxonomic Profile Analyses

Microbiome taxonomic profiling was performed using MetaPhlAn2 software[3] and a proprietary updated version of the MetaPhlAn2 genomic database containing ~1.3 million genomic markers representing 13,400 bacterial and archaeal species. The update includes markers from an expanded set of spore-forming species genomes from public databases and Seres’ strain isolates [4]. For taxonomic profiling, WMS pre-processed data were subsampled to 163,000 mapped reads to ensure a similar limit of detection across samples. Samples with fewer than 163,000 mapped reads were excluded from taxonomic analysis.

The taxonomic profiling output is the relative abundances of species detected. For higher level taxonomic groups (e.g., genus, family), relative abundance of each group was defined as the sum of the relative abundances of species contained within that group.

## Antimicrobial Resistance Gene Profiling

ARG profiling was performed using ShortBRED version 0.9.5[5] and the Comprehensive Antibiotic Resistance Database (CARD) version 3.1.3[6]. A ShortBRED marker database was created with the protein sequences from the CARD protein homolog models, which are organized based on the CARD Antibiotic Resistance Ontology (ARO), where each ARO corresponds to a single distinct ARG.

As ShortBRED has built in read depth and gene length normalization as part of its output, non-subsampled pre-processed WMS data from ECOSPOR III and the healthy cohort were used in the ARG analyses. The data was analyzed using ShortBRED with the ‘shortbred_quantify.py’ command line tool and usearch version 8.0.1623 [7] and using a 90% identity cutoff. The ShortBRED output, per sample, was normalized counts per ARG, represented in reads per kilobase million (RPKM).

ARG normalized counts were “rolled up” (i.e., summed into higher order groups) into gene families, drug class resistances, and resistance mechanisms by summing up all ARGs within each CARD category. Finally, total ARG abundance (in RPKM) was calculated as the sum of normalized counts across all ARGs for a given sample.

## Taxon-ARG correlation analyses

Taxon-ARG correlation analyses were conducted using all available samples across timepoints. Relative abundance of bacterial families within Proteobacteria were correlated with ARG abundances for each antibiotic drug class and resistance mechanism independently, and adjusted using the Benjamini-Hochberg method (corrected once for drug class and separately for resistance mechanism). Firmicutes species were classified as spore-formers or non-spore-formers according to a proprietary, curated annotation of species experimentally demonstrated to form spores or identified in the literature. If at least 50% of species within a given family were classified as spore-formers, it was considered a spore-forming family. The list of spore and non-spore forming families are provided in the **Figure 4** legend.

**Healthy Cohort**

Healthy adults (n=68) were identified and screened via a Seres Therapeutics clinical study stool donor program from a single site (Orange County Research Center) from 2017-2018. Inclusion criteria included age 18–50 years old, BMI 18.5–30, and no oral, intravenous, or intramuscular antibacterial exposure within the last 12 weeks and no antifungal, antiviral, or antiparasitic within the past 8 weeks. All participants provided informed consent prior to screening and stool collection. Single stool samples were collected from each participant at a single timepoint. All sample collection and analysis procedures were the same for the healthy cohort as in ECOSPOR III.

The healthy cohort was 56% male, mean age 30 years and had a mean BMI of 23 kg/m^2^.

To compare ECOSPOR III vs healthy cohort taxonomic composition and ARG data, non-parametric Wilcoxon rank-sum tests were performed per timepoint for each arm vs. the healthy cohort. P-values were adjusted for multiple hypotheses using the Benjamini-Hochberg method.

**SUPPLEMENTARY TABLES**

**Supplementary** **Table 1** Adjusted p values reported from Wilcoxon rank-sum tests comparing phylum relative abundances in the VOS and placebo treatment arms in the ECOSPOR III trial at each timepoint vs. the healthy cohort at a single timepoint as reference

|  | **P value, abundance in study arm vs. healthy** | | | |
| --- | --- | --- | --- | --- |
| **Time point** | **Firmicutes** | | **Proteobacteria** | |
|  | **VOS** | **Placebo** | **VOS** | **Placebo** |
| Baseline | < 0.001 | < 0.001 | < 0.001 | < 0.001 |
| Week 1 | 0.007 | 0.021 | < 0.001 | < 0.001 |
| Week 2 | 0.043 | 0.034 | 0.015 | < 0.001 |
| Week 8 | > 0.1 | 0.049 | 0.021 | < 0.001 |
| Week 24 | > 0.1 | 0.002 | > 0.1 | 0.008 |

VOS=VOWST^TM^ Oral Spores

By Week 24, the abundances of Firmicutes and Proteobacteria in the VOS arm were similar to the healthy cohort, whereas placebo-treated patients had elevated levels of Proteobacteria, with a corresponding depletion of Firmicutes at week 24 relative to the healthy cohort (**Supplementary** **Table 1, Figure 1**).

**Supplementary Table 2** Adjusted p values reported from Wilcoxon rank-sum tests comparing the relative abundance of *Enterobacteriaceae* as well as spore- and non-spore-forming Firmicutes in the VOS and placebo treatment arms in the ECOSPOR III trial at each time point vs. the healthy cohort at a single timepoint as reference

|  | **P value, abundance in study arm vs. healthy** | | | | | |
| --- | --- | --- | --- | --- | --- | --- |
| **Time point** | **Proteobacteria** | | **Firmicutes** | | | |
|  | ***Enterobacteriaceae*** | | **Non-spore-forming** | | **Spore-forming** | |
|  | **VOS** | **Placebo** | **VOS** | **Placebo** | **VOS** | **Placebo** |
| **Baseline** | <0.001 | <0.001 | <0.001 | <0.001 | <0.001 | <0.001 |
| **Week 1** | <0.001 | <0.001 | >0.1 | <0.001 | 0.013 | <0.001 |
| **Week 2** | 0.001 | <0.001 | 0.014 | <0.001 | 0.027 | <0.001 |
| **Week 8** | 0.001 | <0.001 | >0.1 | <0.001 | >0.1 | <0.001 |
| **Week 24** | 0.048 | 0.011 | 0.001 | >0.1 | >0.1 | <0.001 |

VOS=VOWST^TM^ Oral Spores

By week 24, VOS-treated patients had similar abundances of spore-forming and non-spore forming Firmicutes compared with the healthy cohort reference. In contrast, placebo-treated patients in the ECOSPOR III trial had lower abundances of spore-forming Firmicutes and increased non-spore-forming Firmicutes when compared with the healthy cohort reference (Supplementary Table 2).

**Supplementary Table 3** ARG abundance over time in VOS and placebo arms in the ECOSPOR III trial and ARG abundance at a single timepoint in the healthy cohort, with results reported from Wilcoxon rank sum tests comparing each treatment arm at each time point with the healthy cohort at a single timepoint as reference.

|  | **ECOSPOR III** | | | | | | **Healthy (N = 68)** |
| --- | --- | --- | --- | --- | --- | --- | --- |
|  | **VOS** | | | **Placebo** | | |  |
| **Time point** | **N** | **Median ± IQR** | **P value vs. healthy** | **N** | **Median ± IQR** | **P value vs. healthy** | **Median ± IQR** |
| Baseline | 74 | 2734 ± 3362 | < 0.001 | 77 | 2969 ±3920 | <0.001 | 699 ± 389 |
| Week 1 | 66 | 853 ± 726 | 0.046 | 68 | 1372 ± 1883 | <0.001 | - |
| Week 2 | 59 | 779 ± 481 | > 0.1 | 83 | 1053 ± 1476 | <0.001 | - |
| Week 8 | 66 | 759 ± 601 | > 0.1 | 56 | 914 ± 859 | 0.024 | - |
| Week 24 | 52 | 785 ± 550 | > 0.1 | 42 | 814 ± 535 | > 0.1 | - |

VOS=VOWST^TM^ Oral Spores

By Week 2, VOS-treated patients had levels of ARGs that were comparable with the healthy cohort and durable through 24 weeks (Supplementary Table 3). In contrast, placebo-treated patients did not reach comparable ARG abundance with the healthy cohort until Week 24.

**Supplementary Table 4** Adjusted p values of the differences of abundance of antibiotic drug classes in VOS and placebo treatment arms across time in the ECOSPOR III trial vs. the healthy cohort at a single timepoint, as reported by independent Wilcoxon rank sum tests

| **Drug class** | **Week 1** | | **Week 2** | | **Week 8** | | **Week 24** | |
| --- | --- | --- | --- | --- | --- | --- | --- | --- |
|  | **Placebo** | **VOS** | **Placebo** | **VOS** | **Placebo** | **VOS** | **Placebo** | **VOS** |
| Aminoglycoside | **<0.001** | **<0.001** | **<0.001** | **0.022** | **<0.001** | **0.005** | **0.008** | **0.012** |
| Beta-lactam | **<0.001** | **<0.001** | **<0.001** | **0.03** | **0.001** | 0.088 | 0.254 | 0.637 |
| Carbapenem | **<0.001** | **<0.001** | **<0.001** | 0.093 | **0.002** | 0.271 | 0.432 | 0.553 |
| Cephalosporin | **<0.001** | 0.088 | **<0.001** | 0.831 | **0.007** | 0.656 | 0.089 | 0.417 |
| Glycopeptide | **<0.001** | **0.046** | **0.001** | 0.277 | **<0.001** | 0.305 | **0.001** | 0.154 |
| Glycylcycline | **<0.001** | **0.005** | **<0.001** | 0.374 | **0.005** | 0.216 | 0.967 | 0.505 |
| Macrolide | **<0.001** | 0.254 | **<0.001** | 0.905 | **0.008** | 0.423 | 0.336 | 0.967 |
| Monobactam | **<0.001** | **<0.001** | **<0.001** | **0.004** | **<0.001** | **0.001** | 0.139 | **0.032** |
| Quinolone | **<0.001** | **<0.001** | **<0.001** | **0.013** | **<0.001** | 0.067 | 0.16 | 0.505 |
| Sulfa | **<0.001** | **0.001** | **<0.001** | **0.005** | **<0.001** | **<0.001** | **<0.001** | **<0.001** |
| Tetracycline | **0.046** | 0.305 | 0.089 | 0.737 | 0.825 | 0.968 | 0.305 | 0.714 |
| Other | **<0.001** | **0.019** | **<0.001** | 0.335 | **0.012** | 0.277 | 0.968 | 0.346 |

VOS=VOWST^TM^ Oral Spores

Compared with the healthy cohort, VOS-treated patients had comparable ARG abundances by Week 8 in most drug classes, while placebo maintained significantly elevated levels at Week 8 in all but tetracycline resistance (Supplementary Table 4). By week 24, both treatment arms had only a few antibiotic class resistances elevated above the healthy cohort.

At weeks 1 and 2, VOS-treated patients also had levels of “antibiotic target protection” which were comparable with levels observed in the healthy cohort.

**SUPPLEMENTARY FIGURES**

**Supplemental Figure 1.**

**
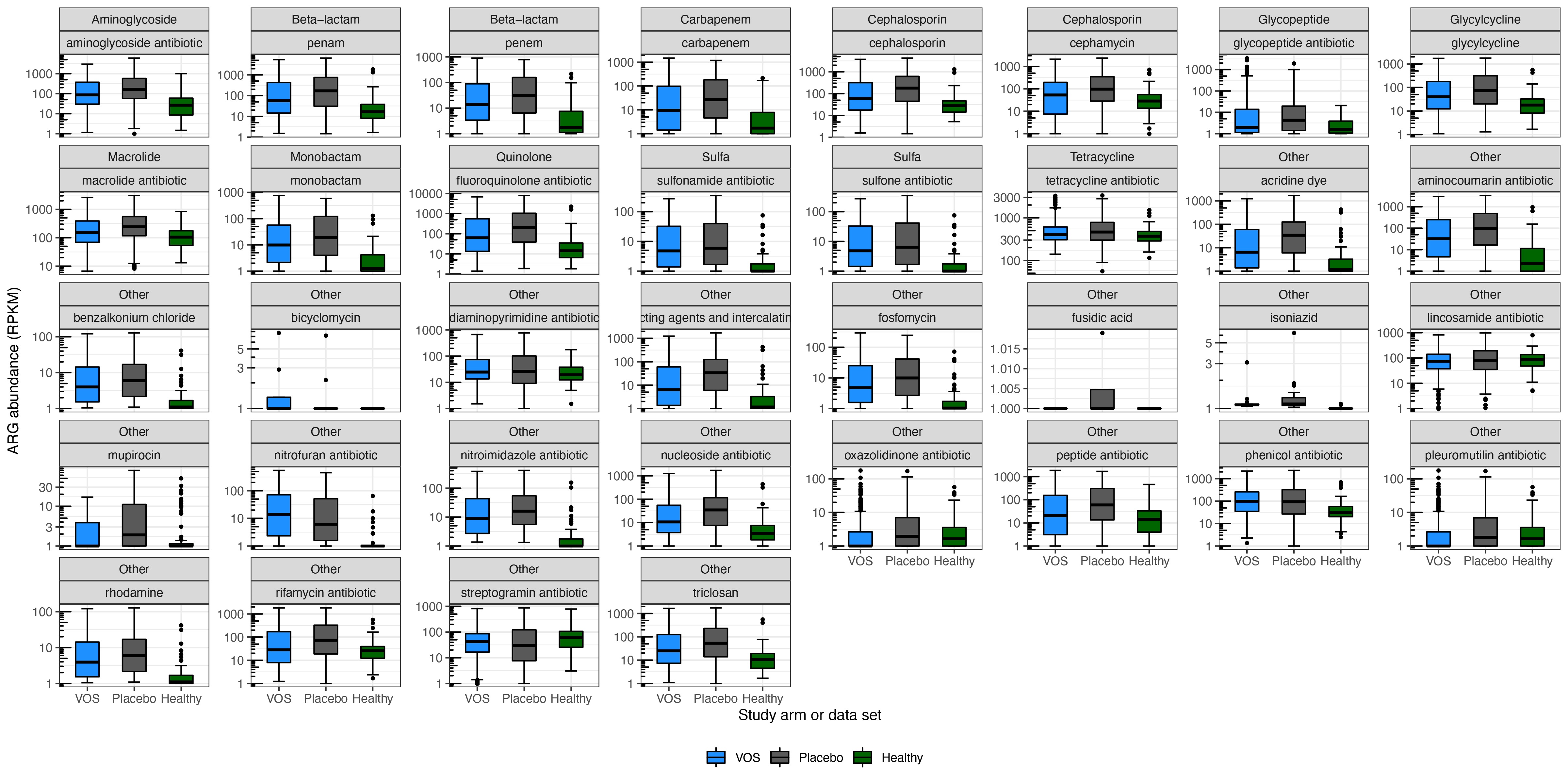
**

**Supplemental Figure 2.**

**
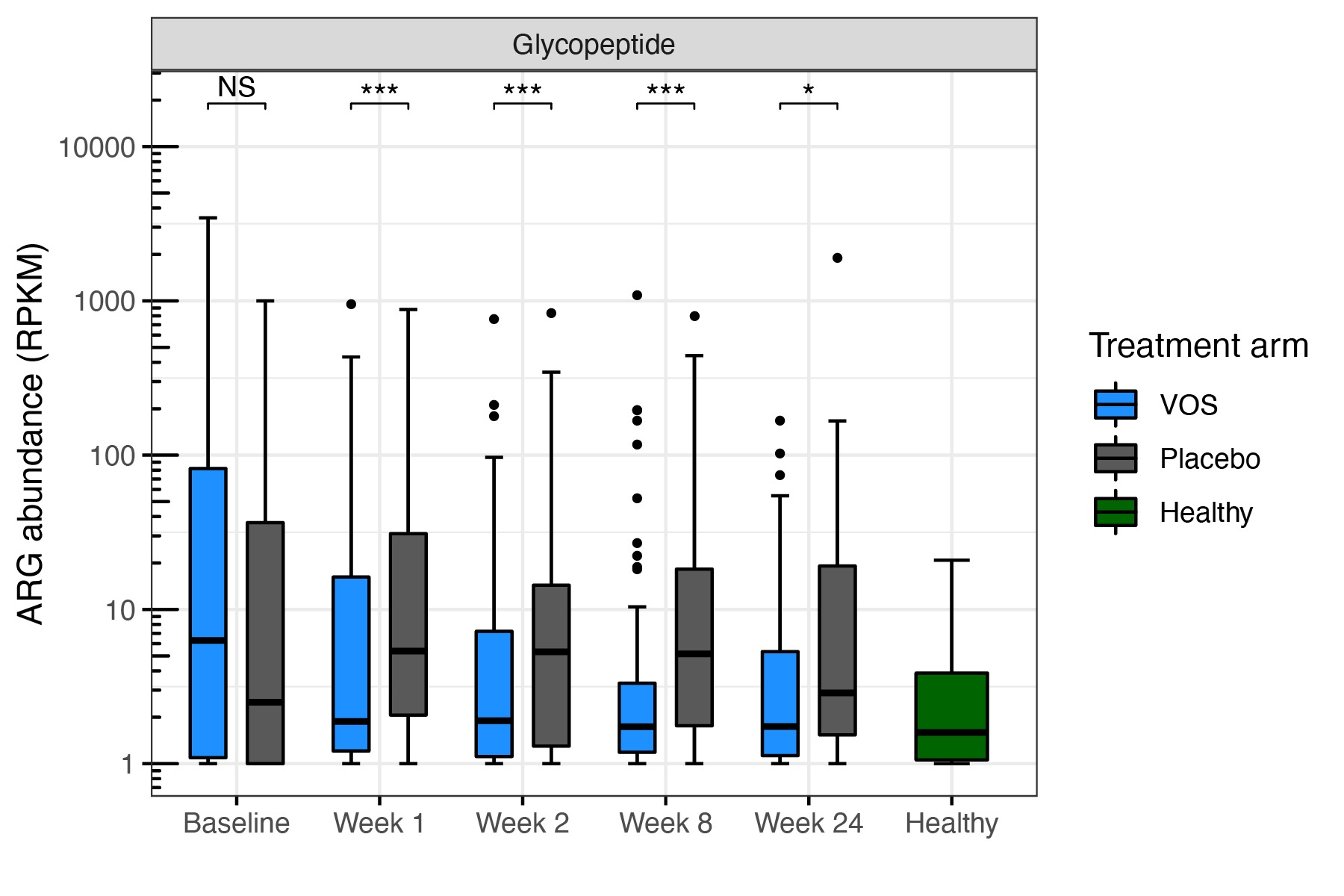
**

**Supplemental Figure 3.**


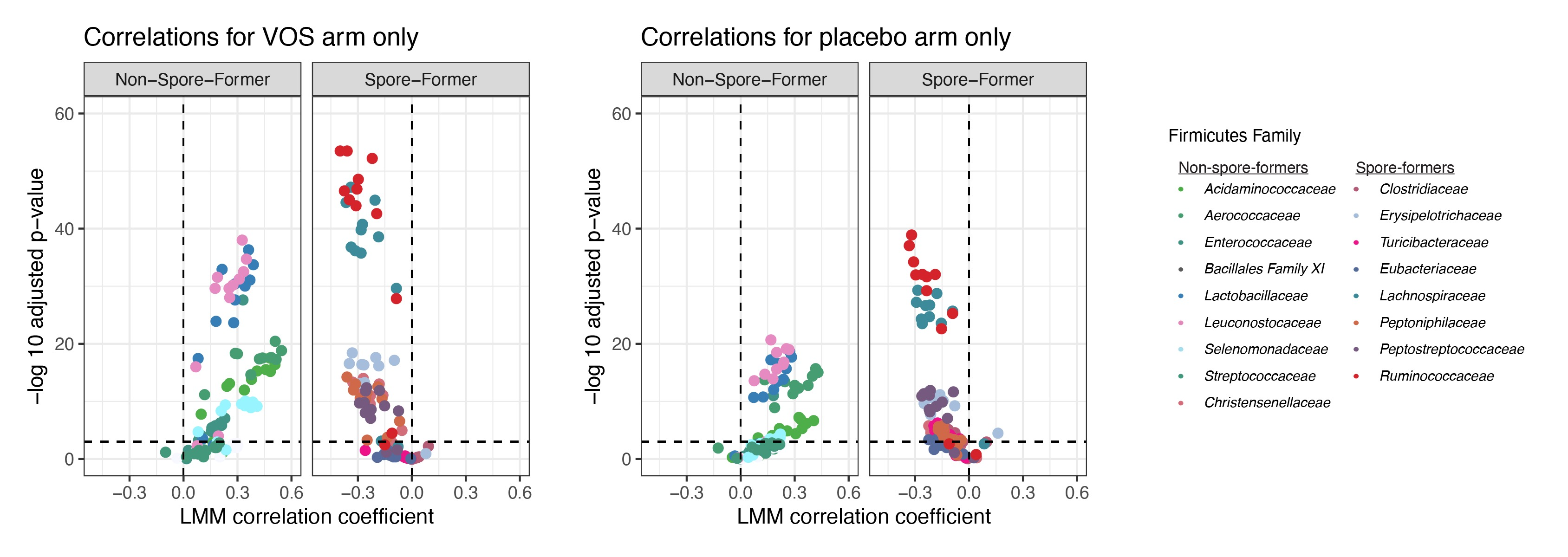


**References**

1. Bolger AM, Lohse M, Usadel B. Trimmomatic: a flexible trimmer for Illumina sequence data. Bioinformatics **2014**; 30:2114–2120.

2. Langmead B, Salzberg SL. Fast gapped-read alignment with Bowtie 2. Nat Methods **2012**; 9:357–359.

3. Truong DT, Franzosa EA, Tickle TL, et al. MetaPhlAn2 for enhanced metagenomic taxonomic profiling. Nat Methods **2015**; 12:902–903. Available at: <http://www.nature.com/doifinder/10.1038/nmeth.3589>.

4. Feuerstadt P, Louie TJ, Lashner B, et al. SER-109, an Oral Microbiome Therapy for Recurrent Clostridioides difficile Infection. New Engl J Med **2022**; 386:220–229.

5. Kaminski J, Gibson MK, Franzosa EA, Segata N, Dantas G, Huttenhower C. High-Specificity Targeted Functional Profiling in Microbial Communities with ShortBRED. PLoS Comput Biol **2015**; 11:e1004557.

6. Alcock BP, Raphenya AR, Lau TTY, et al. CARD 2020: antibiotic resistome surveillance with the comprehensive antibiotic resistance database. Nucleic Acids Res **2019**; 48:D517–D525.

7. Edgar RC. Search and clustering orders of magnitude faster than BLAST. Bioinformatics **2010**; 26:2460–2461.
